# Valuing Acute Health Risks of Air Pollution in the Jinchuan Mining Area, China: A Choice Experiment with Perceived Exposure and Hazardousness as Co-Determinants

**DOI:** 10.3390/ijerph16224563

**Published:** 2019-11-18

**Authors:** Zhengtao Li

**Affiliations:** 1 School of Economics, Zhejiang University of Finance & Economics, Hangzhou 310018, China; zhengtao_li@126.com; 2 Center for Economic Behavior and Decision-Making, Zhejiang University of Finance & Economics, Hangzhou 310018, China

**Keywords:** air pollution, perceived health risk, random parameter logit model, model under-specification, willingness to pay, China

## Abstract

This paper analyzes the choice of illness-cure combinations to estimate people’s willingness to pay (WTP) for the reduction of acute health risks correlated with air pollution caused by mining and smelting in the Jinchuan mining area, China. To improve explaining the power of choice experiment (CE), a random parameter logit model (RPL) was employed and extended by considering rank ordered choice sets and non-linear effects of health risk perception on choice behaviors. The results of this study indicated that the ordered RPL approach produced better results than the unordered alternative after comparing different modeling techniques. Perceived health risk, illness attributes, and the residents’ external characteristics: income, education, age, family health experience, work environment and proximity to pollution source are important determinants of the Jinchuan people’s choice mode for avoiding acute health risks caused by air pollution. Taking all acute illnesses investigated together, the mean Jinchuan household WTP for reducing acute health risk caused by air pollution is 146.69 RMB (abbreviation of Chinese yuan) per year (US$23.38, 0.31% of average yearly household income). On the basis of our findings, we conclude that virtually Jinchuan residents perceive air pollution as a serious health risk. To assist the residents to take appropriate preventive action, the local government should develop counseling and educational campaigns and institutionalize disclosure of air quality conditions.

## 1. Introduction

Outdoor air pollution has become the biggest environmental challenge for Chinese public health [1,2]. Chen et al. [3] found that the average annual exposure to PM_2.5_ in the 272 Chinese cities was 56 μg/m^3^—much above the World Health Organization air quality guidelines of 10 μg/m^3^. Each 10 μg/m^3^ increase in air pollution was respectively associated with a 0.22 percent increase in mortality from all non-accident related causes, a 0.29 percent increase in all respiratory mortality and a 0.38 percent increase in chronic obstructive pulmonary disease (COPD) mortality [3]. 

Jinchuan, which is one of the ten cities with the most polluted air in China [4], has the largest nickel resource and output in China and has been called the nickel capital of China. Mining and smelting industries dominate its economy and make a substantial contribution to its development. Nearly 50% of the workforce is employed in these industries and 70% of the government receipts of the city of Jinchuan derive from them [5]. However, the nickel industries also cause serious air pollution, suspended particles, sulfur dioxide, chlorine gas, and nitrogen dioxide are Jinchuan’s main air pollutants [4,6]. Jinchuan residents suffered from high health risks due to the above four air pollutants. Zheng et al. [7] found that a 10 μg/m^3^ increase of suspended particles, sulfur dioxide and nitrogen dioxide respectively associated with an increase of 0.5%, 0.7% and 3.4% hospital admission of respiratory diseases in Jinchuan mining area. In addition, Jinchuan is a prefecture-level city and local households mainly relied on electricity and gas for cooking. Thus, the main health challenge deriving from air pollution in Jinchuan mining area is outdoor air pollution rather than indoor air pollution.

The main objective of this study is to value Jinchuan residents’ preferences for avoiding acute health risks caused by air pollution by using choice experiment (CE). CE has been used to value people’s preference for avoiding health risks caused by air pollution. Rodriguez and Leon [8] studied the health effects caused by emissions from a large power plant in Las Palmas de Gran Canaria (Spain) and found that people’s preferences are significantly influenced by the magnitude of the reduction of the risk of becoming ill, the duration of illness episodes, and the limitations imposed by the illness. Banfi et al. [9] studied the impact of air pollution externalities on human welfare in Zurich and Lugano by way of CE and found that the willingness to pay (WTP) is positively and significantly related to the pollution reduction level. The mean household WTP for air quality improvement from bad to good in Zurich was 198 CHF and 151 CHF in Lugano. Yoo et al. [10] conducted a CE to quantify the environmental costs of air pollution impacts on mortality, morbidity and poor visibility in Seoul. The author found that an individual’s average monthly WTP is approximately 5494 Korean won (US$4.6) for a 10% reduction of the concentrations of the major pollutants in Seoul’s air (0.23% of per capita monthly income). The number of CE studies to value health risk caused by air pollution, however, is very limited. 

The main advantage of using CE to value health risks caused by air pollution is that it mimics purchase situations by focusing on typical health status attributes such as symptoms of specific diseases and their duration and presenting a set of predetermined choices to consumers to estimate their WTP for health risk reduction [8,9,10]. In addition, the reason why we focus on acute health risks, particularly, acute upper respiratory tract infection is because these symptoms are common and clearly discernible risks of air pollution [11,12]. Furthermore, this feature facilitates respondents’ understanding of the health problem at hand, and thus of making a choice. Bresnahan [13] pointed out that people are more sensitive to take actions against acute health problems than to chronic health impairments.

The health-related CE studies mentioned above commonly explained choice behaviors in terms of illness characteristics like symptom and duration, and residents’ external characteristics such as age, income and education. However, they ignored the role of psychological factors in preference formation (notably perception) which may lead to an inadequate explanation of behavior [14], specifically omitted variable bias [15,16]. Um et al. [17] amongst others, also confirmed that perceived health risks are important predictors of behaviors aimed at reducing health risks caused by environmental degradation. Furthermore, research in other areas, especially transportation research, showed that incorporating psychological factors into choice modeling can significantly improve the explanatory power of the traditional choice model (e.g., Temme et al. [18]). Thus, we include the psychological factor perceived health risk in this CE study, in addition to the conventional explanatory variables, notably illness characteristics and socioeconomic characteristics. 

This study makes two primary contributions to the literature. First, this study contributes to the relevant literature by extending random parameter logit (RPL) through considering psychological factors-notably, perceived health risk. This results in a better understanding of respondents’ decision-making processes [14,15,16,17] and improvement of the explanatory power of the RPL [18]. Second, two dimensions of perceived health risk: exposure and hazardousness were distinguished in this research, and their non-linear effects on choice behavior indicating Jinchuan residents may also use other mechanisms than medicines to reduce acute health risks caused by air pollution, for instance, installing air filters at home or restricting outdoor activities.

## 2. Conceptual Model and Methods

In a CE, the researcher presents two or more hypothetical commodities (a choice set) to the respondents, and asks them to choose the most preferred one. The commodities are described in terms of bundles of attributes. Table 1, based on Johnson et al. [19] and on consultation with local doctors, presents several attributes and levels of acute health risks that are typically correlated with air pollution in Jinchuan. The four attributes are: “illness”, “activity restriction”, “duration” and “price”. The first three attributes are symptoms, whereas the fourth attribute, “price”, is the amount of money that a respondent is willing to pay per time period for the cure (medicines or seeing doctor) to reduce a combination of symptoms. 

In Table 1, for the four attributes with three levels each, there are 81 alternatives. Since it is practically impossible to ask each individual in the sample to evaluate all of the 81 alternatives, the number of alternatives has to be reduced. Sandor and Wedel [20] developed a heuristic search procedure to obtain an efficient CE design. We applied this algorithm which gave 12 choice sets. In the next step, implausible or uninformative alternatives were eliminated which gave 6 choice sets (presented in Appendix A). This is similar to Johnson et al. [19] which contains 8 choice sets. Table 2 illustrates a typical choice set derived from Table 1 after application of the search and elimination procedure. Alternatives A and B are two hypothetical goods a respondent could choose. Specifically, alternative A is a scenario of 5 days of acute pneumonia which restricts a subject’s activities in that they cannot leave their home. If the subject spends 300 RMB per year to purchase medicines or to see a doctor, they can avoid acute pneumonia. For the avoidance of 9 days of acute bronchitis at home the price of avoidance is 500 RMB. Note that subjects can also choose alternative C “I don’t want to purchase either” and instead take the risk. 

As mentioned in Section 1, in standard CE, choices are assumed to be functionally related to residents’ external characteristics such as income and age, and to illness attributes, such as the ones in Table 1 and Table 2. However, as discussed above, we hypothesize that the psychological factor perceived health risk is also an important determinant of behaviors aimed at reducing health risks. Hence, the conceptual mode applied in this study contains the following three categories of variables:

(1) Illness characteristics: i.e., type of illness, duration, activity restriction, and price of prevention (cure).
(2) Perceived health risk: health risk perception on exposure and hazardousness.
(3) Respondent’s external characteristics: including age, family size, income, education, family health experience, work environment and proximity to the pollution source.

### 2.1. Choice Sets

We sequentially presented the 6 choice sets in Appendix A to each respondent, each consisting of 3 alternatives. Choice set 1 is presented in Table 2. In this set the alternatives are ordered: alternative A portrays a relatively mild illness situation with low price to avoid it, alternative B a relative severe condition with higher price while alternative C is the status quo: accept the situation, no purchase of medicines or visits to doctors (reference choice). Respondents are asked to select the most preferred alternative in each choice set. Hence, for each choice set, one alternative is chosen. (See Appendix A). Note that acute respiratory illnesses also strongly correlate with weather condition: e.g., temperature, relative humidity and rain fall [21,22]. To estimate the impacts of air pollution, respondents were told to only consider respiratory impacts additional to weather impacts. 

### 2.2. Illness Characteristics

We assume that the three attributes type of illness, duration and activity restriction are the main choice determinants which influence an individual’s disutility directly or indirectly (via the impacts on the lives of their family members). Hence, respondents have an incentive to choose alternative A or B. We follow Dickie and Messman [23] and Johnson et al. [19] and facilitate comparison of alternatives by combining the Type of illness (illness for short) and duration. In addition, we take the natural log of (duration + 1) which gives the variable illness*ln(duration + 1) [23]. The activity restriction levels have a natural ordering with no limitation representing the best outcome and in hospital the worst. Price is the amount of money people are willing to pay to reduce the acute health risk for a year by purchasing the cure. Hence, there is a tradeoff between price and reducing the illness. For further details, see Dickie and Messman [23] and Johnson et al. [19].

### 2.3. Perceived Health Risk

Following Menon et al. [24], we define perceived health risk as the subjective assessment of risk correlated with one’s health for a specified period of time. We assume that people who have a higher perceived health risk commonly will take more actions to protect themselves [25,26]. Hence, perceived health risk increases the likelihood of purchasing alternative A or B. Perceived health risk includes perception of the susceptibility as well as of the consequences of a negative health outcome [27,28]. Thus, perceived health risk caused by air pollution includes two dimensions [27,28]: (i) perceived health risk caused by the intensity of exposure (exposure, for short) and (ii) perceived health risk caused by the hazardousness of pollutants (hazardousness). In the questionnaire the first dimension was measured by the question: “what, in your perception, was the average number of days per week Jinchuan’s air was heavily polluted during the past year?”. The second dimension was measured by the question: “how much health risk do you think Jinchuan’s air pollution poses to you and your family members. Answer this question on a scale from one to ten, where one is the lowest high risk and ten the highest”.

### 2.4. Respondent’s External Characteristics

Following the CE literature [14,18,19], we assume that residents’ external characteristics also impact on their choice behavior. In this paper, age, income, education, family size, family health experience, work environment and proximity to the pollution source are included in the analysis because each has been shown to be a significant predictor of choice behavior aimed at reducing negative health effects caused by environmental degradation. We distinguish three work environment classes: (1) MS (miners and smelter workers of Jinchuan Mining Company (JMC)), (2) NMS (JMC, but not miners or smelter workers) and (3) NMC (non-JMC individuals) which is the base case. In addition, we also distinguish three proximity categories: (1) SAP (close to the smelting plants, serious air pollution), (2) MAP (medium air pollution) and (3) LAP (far away from the source, light air pollution) which is the reference case.

### 2.5. The Random Parameter Logit Model (RPL)

The random utility model is the standard approach to analyze choice experiment responses [29,30,31]. In the random utility framework individual *i*’s (*i* = 1, …, *N*) utility associated with alternative *j* (*j* = 1, …, *J*) in choice set *m* (*m* = 1, …, *M*) is given by

(1) $$U_{ijm}=bs_{ijm}+\gamma z_{i}+c\eta_{i}+\nu_{ijm}$$

with choice

(2) $$d_{ijm}=\{\begin{array}{llll} 1 & \mathrm{if} & U_{ijm}\;\geq U_{ikm} & j,k\in R_{m}\\ 0 & & \mathrm{otherwise} & \end{array}$$

where for respondent *i* $s_{ijm}$ is the (*s* × 1) attribute vector (in the present study $s_{ijm}$ consists of the elements (illness*ln(duration + 1)), activity restriction, Price) of alternative *j* in choice set m, $z_{i}$ is the (*g* × 1) vector of observable characteristics (e.g., age, family size, etc.), $\eta_{i}$ the (*p* × 1) vector of psychological variables (exposure and hazardousness), $\nu_{ijm}$ the error term that follows an extreme-value (Weibull) distribution. **b**, **c** and γ are (1 × *s*), (1 × *p*) and (1 × *g*) row vectors of unknown coefficients of $s_{ijm}$, $z_{i}$ and $\eta_{i}$, respectively. Equation (2) indicates that respondent *i* chooses alternative *j* from choice set m containing $R_{m}$ alternatives, if and only if the alternative *j* yields higher utility than alternative *k*. 

To estimate model (1)–(2), it is assumed that the error terms $\nu_{ijm}$ of the alternatives in a choice set are distributed independently from each other, i.e., the independence of irrelevant alternatives (IIA) assumption [32,33]. Specifically, the IIA implies that the ratio of choice probabilities between two alternatives in a choice set is unaffected by changes in that choice set. This strong assumption is likely to be violated in practice. The problem can be resolved by applying the random parameter logit model (RPL), which allows the parameters associated with alternative-specific attributes to vary randomly across individuals [34]. Specifically:

(3) $$b=\beta +\omega_{i}$$

where β is the population mean, and $\omega_{i}$ the stochastic deviation that represents individual taste relative to the average taste in the population.

Combining Equations (1) and (3) gives:

(4) $$U_{ijm}=\left(\beta +\omega_{i}\right)s_{ijm}+\gamma z_{i}+c\eta_{i}+\nu_{ijm}=\beta s_{ijm}+\gamma z_{i}+c\eta_{i}+\omega_{i}s_{ijm}+\nu_{ijm}$$

From Equation (4) it follows that the error term $\omega_{i}s_{ijm}+\nu_{ijm}$ is correlated over the attributes of the alternative because of the presence of $\omega_{i}$. We take the coefficient of the attribute price fixed for the following reasons. First, as pointed out by Revelt and Train [34] and Hajivassiliou et al. [35], allowing all coefficients of alternative specific attributes to vary tends to render the RPL model unstable and identification of the model empirically difficult. Specifically, when the stochastic part of utility $\nu_{ijm}^{\prime }=\omega_{i}S_{ijm}+\nu_{ijm}$ in Equation (4) is dominated by $\omega_{i}s_{ijm}$, the error term $\nu_{ijm}$ will have little influence on utility. At the extreme, the error term $\nu_{ijm}$ has no influence on utility (variance of $\nu_{ijm}$ is zero). Consequently, the scaling of utility by the variance of $\nu_{ijm}$ will become unstable and additional scaling is need. Secondly, the marginal willingness to pay (WTP) for another attribute than price is the ratio of that attribute’s coefficient and the coefficient of price. When the latter coefficient is fixed, the distribution of the marginal WTP simply follows the distribution of the attribute’s coefficient. If the coefficient of price also varies, the distribution of the marginal WTP becomes complicated. Therefore, Train [29] suggests to keep the coefficient of *Price* fixed. For further details we refer to Lusk and Schroeder [36], Revelt and Train [34].

Following Johnson et al. [19] and Bech and Gyrd-Hansen [37], we effect-coded the attributes illness* ln(duration + 1) and activity restrictions. Similar to dummy coding, effect coding transforms attributes with, say, H qualitative levels into H-1 dummy variables. Unlike dummy coding, however, effect coding assigns a value −1 rather than 0 to each category for the reference level. For example, in effect coding, gender, which is a two-level variable, is coded as 1 (e.g., for females) and −1 (for males). The coefficients of an effect-coded dummy variables represent the deviation of the category’s mean from the overall or “grand mean” across categories [38]. For example, the coefficient of “in hospital” (effect coded −1) represents the deviation of the mean disutility of “in hospital” from the mean disutility of activity restrictions (mean disutility across levels including “no limitation”, “at home” and “in hospital”). The coefficient for the omitted category is the negative sum of the coefficients for the included categories. There are no guidelines for choosing the omitted category in effect coding [38]. Acute upper respiratory tract infection (denoted AI*LD) and no limitation (denoted NL) were chosen as the omitted categories. Consequently, in terms of Equation (4), the choice RPL model reads as follows:

(5) $$\begin{array}{cc} U_{ijm}=(\beta_{AB*LD} & +\omega_{AB*LD-i})AB*LD_{ijm}\\ & +\left(\beta_{AP*LD}+\omega_{AP*LD-i}\right)AP*LD_{ijm}+\;\left(\beta_{AH}+\;\omega_{AH-i}\right)AH_{ijm}\\ & +\left(\beta_{IH}+\omega_{IH-i}\right)IH_{ijm}+\;b_{price\;}Price_{ijm}+\;c_{1}Exposure_{i}\\ & +c_{2}Exposure_{i}^{2}+c_{3}Hazardousness_{i}+c_{4}Hazardousness_{i}^{2}\\ & +c_{5}Exposure_{i}\times Hazardousness_{i}+\gamma_{1}Family\;size_{i}+\gamma_{2}Income\\ & +\gamma_{3}Education_{i}+\gamma_{4}MAP_{i}+\gamma_{5}SAP_{i}\\ & +\gamma_{6}NMS_{i}+\gamma_{7}MS_{i}+\gamma_{8}Family\;health\;experience{\;}_{i}+\gamma_{9}{Age}_{i}\\ & +\nu_{ijm} \end{array}$$

where *i* refers to individual *i*, AB*LD = acute bronchitis*ln(Duration + 1), AP*LD = acute pneumonia*ln(duration + 1), AH = at home, IH = in hospital. The subscripts of ω (e.g., AH-*i*) indicate individual *i*’s preference for the corresponding attribute level. The two dummy variables serious air pollution area (SAP) and medium air pollution area (MAP) capture proximity to the pollution source with lightly polluted area as the base case. Work environment is also modeled by means of two dummy variables: JMC employees, but not miners and smelter workers (NMS), and JMC miners and smelters workers (MS). Non-JMC employee is the base case. Proximity to the pollution source and work environment were developed to measure Jinchuan residents’ air pollution exposure level (objective measures of air pollution) in living and working places, respectively. Exposure, hazardousness, squared-exposure and squared-hazardousness are included to allow for non-linear effects. Moreover, the interaction term exposure × hazardousness is also included to allow both variables to depend on each other. 

The WTP of individual *i* for the reduction of a specific illness can be obtained from the estimated parameters as follows [32,39]

(6) $$WTP_{i}=b_{price}^{-1}\ln\left\{\frac{\exp\left(\mu V_{i}^{1}\right)}{\exp\left(\mu V_{i}^{0}\right)}\right\}$$

where $b_{price}$ is the coefficient of price indicating the marginal utility of money and μ is a scale parameter which is inversely proportional to the standard deviation of the error distribution. In addition, V is the deterministic component of the utility function with $V_{i}^{0}$ the utility of the initial state and $V_{i}^{1}$ the utility of the alternative state. 

### 2.6. Survey and Descriptive Statistics

**Figure 1:**
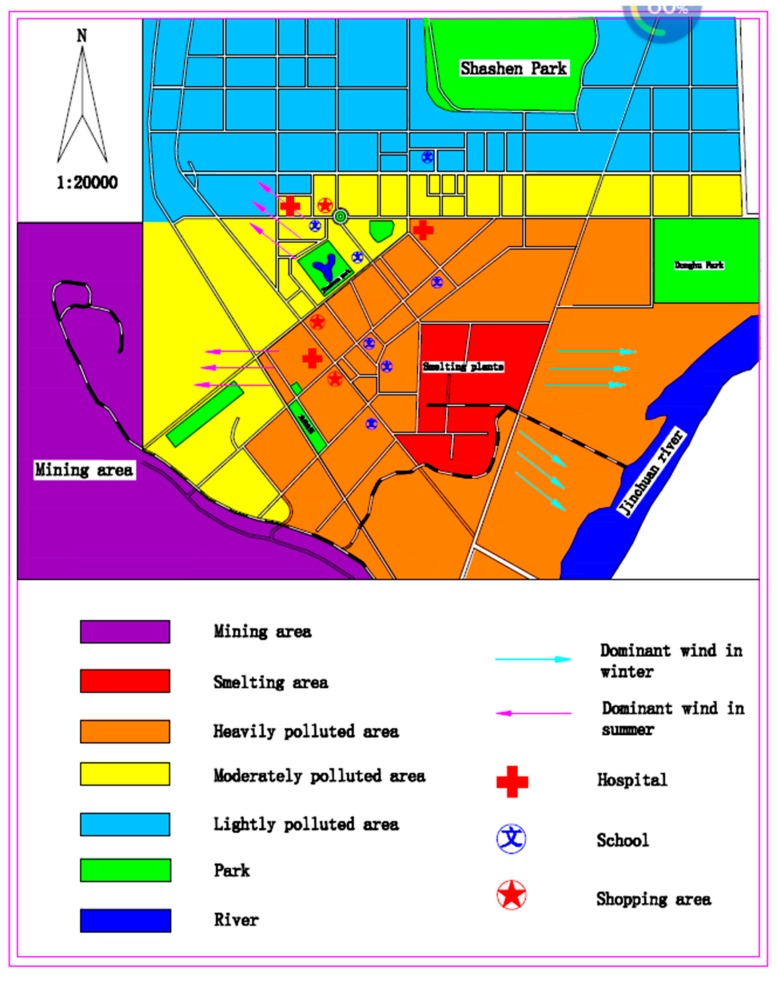
Heavily, moderately and lightly polluted areas of the Jinchuan mining area. Note: the dominant wind directions are from the east and south-east during summer and from west and north-west during winter. Source: JEQMR [40], Wei [4] and Li et al., [41].

Data was collected using a face-to-face survey in the city of Jinchuan, Gansu province, China, in August 2012. A stratified-random sample of 800 respondents between 20 and 80 were selected and interviewed at home. Specifically, the data were collected in two steps. First, the Jinchuan mining area was divided into three sub-areas based on the level of air pollution (corresponding to the distance from the smelting plant): severely polluted, moderately polluted and lightly polluted [4,40,41] (see Figure 1). Secondly, the interviewees in each area were randomly selected in proportion to its total population size. In particular, per hundred households, 1–2 households were randomly selected. The questionnaire contained questions about the respondent’s external characteristics, their perception of exposure and hazardousness, and their choice modes of reducing acute health risks. The questions or outcomes are presented in notes following Table 3.

Of the 800 questionnaires filled out, 41 (5.12%) were rejected because they were incomplete. There was no evidence of non-random drop out. Descriptive statistics are presented in Table 3. 

The distribution of the external characteristics in Table 3 is consistent with Jinchuan’s population distribution [5].

## 3. Empirical Results

As the first step, we estimated Equation (5) based on the 6 choice sets in Appendix A as an unordered RPL model. Fox et al. [42] discussed a general framework for identification of the random parameter logit model and pointed out that the distribution of random coefficients in RPL is nonparametrically identified. The hypothetical illness characteristics (alternatives) in our research, however, are inherently ordered (for instance, severe illness goes together with higher prevention price). Fox [43] pointed out that when respondents select a choice from an unordered subset, the choice probabilities in the subset are rank ordered by the deterministic payoffs. To take the rank order into account, we followed Abdel-Aty [44] and O’Donnell and Connor [45], ordered the choice sets (see Appendix B) and estimated an ordered RPL. Specifically, in Appendix B alternative A in each choice set has low price and describes a relatively mild illness situation; alternative B portrays a relative severe condition with higher price, alternative C is the status quo (reference choice).

Table 4 shows that the ordered RPL model with exposure and hazardousness produces better results than the unordered alternative model. Specifically, its goodness-of-fit measure (McFadden R square) is higher and more of its predictors are significant. Comparison of the ordered-RPL models with and without hazardousness and exposure (Table 4) shows that the psychological variables have explanatory power as measured by the difference in Log-likelihood between both models ($△\chi^{2}=56.7$, df = 10, *p* < 0.001). Comparison of both models furthermore shows that omission of the psychological variables affects the coefficients of the external characteristics, as expected because of under-specification. 

The negative coefficients of AB*LD and AP*LD indicate that compared to the overall mean of illness*ln(duration + 1), AB*LD and AP*LD have more disutility. AI*LD is the omitted category and its coefficient is the negative sum of the included categories [32]. The coefficients of activity restriction monotonically decrease and indicate that higher levels of activity restriction lead to higher utility losses. The coefficient of at home (AH) is insignificant, indicating that it is not different from the overall mean of activity restrictions effect. The coefficient of price is significant and positive indicating that respondents assume that purchasing medicines improves utility. The standard deviation of AP*LD and IH are significant, indicating that there is preference heterogeneity across the respondents. Moreover, these standard deviations are larger than the corresponding means indicating that there is considerable variation across observations that is not explained in the model [19]. 

Table 4 shows that hazardousness and squared hazardousness are both significantly and negatively correlated with purchasing alternatives A and B. An inverted-U curve for exposure was fond. That is, for increasing exposure the possibility of purchasing alternatives A and B initially increases but falls beyond the turning point—one day—when people turn away from the option to reduce health risk through purchasing alternatives A and B, and opt for other preventive actions. This outcome beyond the turning point is in line with the results for hazardousness. Finally, the interaction term exposure × hazardousness significantly increase the possibility of purchasing alternatives A and B indicating that hazardousness and exposure reinforce each other.

We now turn to the external characteristics. The impacts of education on the decision of purchasing alternative A or B are positive and statistically highly significant. This outcome indicates that people with better education tend to take more actions to avoid negative effects of air pollution by purchasing alternative A or alternative B. The coefficient of $\mathrm{Income}{\;}_{A}$ is negative but insignificant indicating that mild health risk does not induce people to take preventive action. The coefficient of $\mathrm{Income}{\;}_{B}$ on the other hand is positive and significant suggesting that people with higher income tend to purchase alternative B to avoid sever illnesses. Family health experience also positively influences the decision to choose alternative A or B rather than C with a slight preference for the more expensive alternative B. The coefficients of age are negative and highly significant indicating that elderly people are more likely to choose alternative C. 

Table 4 furthermore indicates that people who live in an area with medium air pollution are more likely to choose alternative A or alternative B compared to those who live in lightly polluted areas. Furthermore, ${\mathrm{SAP}}_{A}$ is positive, though insignificant whereas ${\mathrm{SAP}}_{B}$ is negative and significant. The latter result indicates that compared to those who live in lightly polluted areas, people in seriously polluted areas prefer to choose alternative C. Table 4 also shows that compared to non-JMC employees, people working in JMC prefer to purchase the more expensive alternative B. For alternative A, the effects of both NMS and MS are insignificant. Finally, the coefficients of family size are negative and significant. 

We also examined heterogeneous effects of perceived health risk on choice behavior by proximity to the pollution source. Table 5 suggests that exposure and squared exposure are highly significant in lightly and moderately polluted neighborhoods but not in severely polluted areas. Similar results hold for hazardousness and squared hazardousness, and hazardousness × exposure, though several of their coefficients in columns 2 and 4 are insignificant, especially for moderately polluted neighborhoods. That is, people who live in the nearby of smelting plants all have high level of risk perception resulting little variation of health risk perception and large standard errors [46].

Based on the above results, we estimate the mean WTP for reducing acute health risk correlated with air pollution by means of Equation (6). For example, the average WTP for avoiding 5 days of acute pneumonia which confines respondents to their home is:

(7) $$\begin{array}{c} WTP_{\mathrm{for}\;\mathrm{avoiding}\;\mathrm{acute}\;\mathrm{pneumonia}\;\left(5\;\mathrm{days}\;\mathrm{at}\;\mathrm{home}\right)}\\ =\frac{\mathrm{Utility}\;\mathrm{of}\;\mathrm{avoiding}\;\left(\mathrm{acute}\;\mathrm{pneumonia}(5\;\mathrm{days}\;\mathrm{at}\;\mathrm{home}\right)-\mathrm{Utility}\;\mathrm{of}\;\mathrm{keeping}\;\mathrm{status}\;\mathrm{quo}\;}{\mathrm{marginal}\;\mathrm{uility}\;\mathrm{of}\;\mathrm{money}\;}\\ =\;\frac{-(\ln\;\left(5+1\right)\times \beta_{\mathrm{AP}*\mathrm{LD}}+\beta_{\mathrm{at}\;\mathrm{home}})-0}{b_{\mathrm{price}}}\\ =\frac{-\left(1.7916\times \left(-0.049\right)+\left(-0.040\right)\right)}{0.037}=3.468\;\mathrm{RMB} \end{array}$$

We estimated the average WTP for the three illness*ln(duration + 1). The results are presented in Table 6. The third column shows zero WTP in the case of no limitation for the three acute respiratory illnesses. This outcome indicates that these health problems are seen as so minor that spending money to avoid them does not really increase utility. The fifth column shows the WTP estimates for the in hospital restriction. First, note that there is no WTP estimate for the combination acute upper respiratory tract infection and in hospital because hospitalization for this kind of illness is rare. Secondly, for the other diseases the results show that average WTP increases by duration. In addition, the WTP for avoiding hospitalization is larger than for the activity restriction at home. This result is consistent with Johnson et al. [19]. Taking all the illnesses investigated in Table 6 together gives a mean household WTP equal to 146.69 RMB per year (US$23.38, 0.31% of average yearly household income).

## 4. Discussion

This paper analyzed people’s choice of illness-cure combinations to estimate their willingness to pay (WTP) for the reduction of acute health risks correlated with air pollution caused by mining and smelting in the Jinchuan mining area, China. This study firstly contributes to the relevant literature by extending RPL through considering rank ordered choice sets. We found that the ordered RPL approach produced better results than the unordered alternative after comparing different modeling techniques, and this is consistent with Abdel-Aty [43] and O’Donnell and Connor [44]. Hence, the ordered RPL approach is recommended to model choice when the alternatives are ordered.

In addition, as mentioned in the introduction part, psychological factors are not routinely considered in evaluation studies including choice modeling studies. However, their omission can lead to under-specification and biased estimation (omitted variable bias and inflated error variance). Thus, Jinchuan residents’ perception of the intensity of exposure to air pollution and of hazardousness of pollutants were included in RPL to avoid above two issues in this study. In fact, health risk perception is an important vehicle to raising awareness which in its turn affects behavior [25,26]. This is also confirmed in our research, and we found exposure and hazardousness both significantly impact Jinchuan people’s choice behaviors and induce them to take the right kind of averting action to avoid health risks caused by air pollution. Moreover, the explaining power of conventional RPL was significantly improved in our study by taking non-linear effects of perceived health risk into account. This is in line with Temme et al. [18] and Nauges et al. [24]. In future, more research efforts should be made to understand the omitted variable bias caused by ignoring psychological factors in choice modelling studies.

Another important finding is that both kinds of perceived health risk: exposure and hazardousness significantly and non-linearly influence Jinchuan resident’s choice behavior indicating that residents may also use other mechanisms than medicines to reduce health risks caused by air pollution. Specifically speaking, negative impacts of perceived health risk on the propensity to buy alternatives A and B do not imply that they are not important determinants of people’s preferences to avoid diseases correlated with air pollution in general. The results rather show that people with higher perception of hazardousness do not view medicines or seeing a doctor as an appropriate strategy to reduce the disease risk of acute respiratory diseases, they may opt for other preventive actions than buying medicine or seeing a doctor, for example, installing air filters at home or spending more time indoor. See also Johnson et al. [19] and Tsuge et al. [11].

Understanding the choice illness-cure combination is useful information for policy makers in the Jinchuan mining area for the development of environmental policies in the long run. The results indicate that health concerns are major drivers of people’s behavior. Therefore, improving air quality ought to be a major long run policy objective. As it takes time to implement air quality improving policies, a short run disclosure policy should be installed to provide the inhabitants information to take the right actions, particularly medicines, to reduce health risks.

The present study can be extended in several additional ways. First, the illnesses considered in this paper are acute upper respiratory tract infection, acute bronchitis and acute pneumonia. Note that the focus on these acute health risks implies a limitation on the estimated willingness to pay (WTP) because of the omission of other kinds of diseases, notably non-acute diseases. Apart from acute health risk, CE also can be used to value people’s preference for avoiding chronic health risk and premature mortality correlated with air pollution. Another restriction is that only the use of medication is considered while other types of averting behaviors such as restricting outdoor activities may also be taken. The WTP estimated by means of CE in this paper thus provides a lower bound of the total WTP. Despite the useful findings of this study for academia and practical applications, its limitations should be recognized. As the proposed research model in this study was only tested with Jinchuan mining area residents, the findings of this study may not be generalized to other regions due to natural and cultural differences. This study should be replicated with other populations to verify and generalize the research findings in the future.

## 5. Conclusions

This study is the first to provide insights into Jinchan residents’ choice behavior for avoiding acute health risks caused by mining and smelting industries. Illness attributes were type, duration and activity restriction while price was the main cure characteristic. The illnesses considered were acute upper respiratory tract infection, acute bronchitis and acute pneumonia. In addition to the attributes of the illness-cure combination and the conventional external characteristics, perceived health risk due to (i) intensity of exposure to polluted air and (ii) hazardousness of pollutants were taken into account as determinants of choice. Results showed the illness attributes, and the external characteristics: income, education, age, family health experience, work environment and proximity to pollution source were the important determinants of choice mode. In addition, exposure and hazardousness significantly influence Jinchuan residents’ choice behavior. Particularly, the inverted-U shape between exposure and possibility of purchasing alternatives A and B was observed. Moreover, interaction term of exposure and hazardousness significantly impact Jinchuan residents choice behavior indicating that the mean effect of hazardousness is enhanced by people with higher exposure. Moreover, given the findings of this study, practical policy implementation for reducing health risks caused by air pollution were discussed earlier. 

## Figures and Tables

**Table 1:** Attributes and attribute levels.

Attribute	Levels of Attributes	Description
Illness	(1) Acute upper respiratory tract infection	Sneezing, a runny nose, cough and fever
	(2) Acute Bronchitis	Cough, fever, burning or dull pain in the chest, wheezing.
	(3) Acute Pneumonia	Chest pain, fever, and difficulty breathing.
Duration (days)	(1) 5	5-day episode
	(2) 9	9-day episode
	(3) 15	15-day episode
Activity Restriction	(1) No Limitation	No physical limitations nor restrictions of activities
	(2) At home	Stay in the house, without social or recreational activities
	(3) In Hospital	In hospital and help needed to take care of oneself
Price of cure (annual)	(1) 100 RMB	
	(2) 300 RMB	
	(3) 500 RMB	

Based on Johnson et al. [19] and consultation with local doctors.

**Table 2:** A typical choice set.

	Alternatives	A	B	C
Attributes				
Disease		Acute Pneumonia	Acute Bronchitis	I don’t want to purchase either
Duration		5-day episode	9-day episode	
Daily activity restriction		At home	At home	
Price of cure (annual)		300 RMB	500 RMB	
Which alternative do you prefer to purchase				

**Table 3:** Descriptive statistics for the observed exogenous variables.

Variables	Min	Max	Mean	S.D.
**Age**	21	78	44.11	11.4
**Family size**	1	6	3	0.78
**Family health experience**	0	1	0.33	0.48
**Exposure**	0	7	2	1.53
**Hazardousness**	1	10	7.46	1.51
**Education**	%	**Work environment**		%
Primary school	6.30%	Non-JMC employee (reference case)		59.55%
Middle school	23.60%	Miners and smelter workers of JMC (MS)		18.18%
High school	25.30%	JMC employee, but not miner or smelter worker (NMS)		22.27%
Vocational school	25.30%	**Household Income (RMB per month)**		%
Bachelor’s degree	19.10%	1000–2000		4.70%
Master’s degree	0.40%	2000–3000		15.30%
		3000–4000		18.30%
**Proximity to the pollution source**	%	4000–5000		19.10%
Nearby smelting plants, severe air pollution (SAP)	29.60%	5000–6000		20.90%
Medium distance, medium air pollution (MAP)	29.80%	6000–7000		13.00%
Far away from smelting plants, light air pollution (LAP)	40.60%	More than 7000		8.60%

**Note: Family size:** number of family members living in the same house. **Family health experience:** 1 if the respondent or one or more of their family members have been hospitalized for cardiovascular diseases (e.g., hypertension, heart attack, chest pain, arrhythmia and myocardial infraction) or respiratory diseases (e.g., upper respiratory tract infection, bronchitis, pneumonia, asthma, and lung cancer), 0 otherwise. Source: Author’s survey.

**Table 4:** The estimated RPL models.

1	2	3	4	5	6	7
	Ordered-RPL Model with *Exposure* and *Hazardousness*		Ordered-RPL Model without *Exposure* and *Hazardousness*		Unordered-RPL Model with *Exposure* and *Hazardousness*	
Variables	Coefficient	T-Value	Coefficient	T-Value	Coefficient	T-Value
Acute upper respiratory tract infection * ln(duration + 1) (AI*LD)	0.106	-	0.101	-	−0.047	-
S.D.	-	-	-	-	-	-
Acute Bronchitis * ln(Duration + 1) (AB*LD)	−0.057 **	−2.067	−0.061 **	−2.205	−0.038	−1.381
S.D.	0.000	0.000	0.000	0.000	0.000	0.000
Acute Pneumonia * ln(Duration + 1) (AP*LD)	−0.049 *	−1.722	−0.050 *	−1.757	0.009	0.354
S.D.	0.489 ***	13.788	0.481 ***	13.508	0.445 ***	12.720
No Limitation (NL)	0.661	-	0.715	-	0.698	-
S.D.	-	-	-	-	-	-
At home (AH)	−0.040	−0.069	−0.040	−0.073	0.087	0.151
S.D.	0.000	0.000	0.000	0.000	−0.002	0.000
In hospital (IH)	−0.621 ***	−5.874	−0.675 ***	−6.098	−0.611 ***	−5.605
S.D.	1.545 ***	8.618	1.607 ***	8.889	1.622 ***	9.551
*Price*	0.037 ***	5.383	0.037 ***	5.490	0.007 **	2.130
$Exposure_{A}$	0.226 ***	4.934			0.218 ***	4.623
$Exposure_{B}$	0.175 ****	4.238			0.190 ***	4.520
$Exposure_{A}^{2}$	−0.147 ***	−9.583			−0.148 ***	−9.289
$Exposure_{B}^{2}$	−0.085 ***	−6.079			−0.125 ***	−8.857
$Hazardousness_{A}$	−0.134 ****	−3.623			−0.108 ***	−2.865
$Hazardousness_{B}$	−0.086 ***	−2.580			−0.091 ***	−2.726
$Hazardousness_{A}^{2}$	−0.044 ***	−3.178			−0.030 **	−2.144
$Hazardousness_{B}^{2}$	−0.017	−1.327			−0.024 *	−1.853
$Exposure\times Hazardousness_{A}$	0.139 ***	6.251			0.087 ***	3.961
$Exposure\times Hazardousness_{B}$	0.086 ***	4.406			0.046 **	2.465
$Family\;{health}_{A}$	0.161	1.503	0.117	1.096	0.308 ***	2.882
$Family\;{health}_{B}$	0.563 ***	5.613	0.533 ***	5.352	0.434 ***	4.438
$Family\;{size}_{A}$	−0.342 ***	−5.039	−0.296 ***	−4.346	−0.357 ***	−5.200
$Family\;{size}_{B}$	−0.294 ***	−4.583	−0.256 ***	−4.016	−0.333 ***	−5.340
${Income}_{A}$	−0.056	−1.123	−0.077	−1.553	−0.012	−0.243
${Income}_{B}$	0.097 **	2.152	0.082 *	1.842	0.030	0.682
${Education}_{A}$	0.259 ***	7.207	0.243 ***	6.968	0.305 ***	8.605
${Education}_{B}$	0.396 ***	11.557	0.386 ***	11.522	0.355 ***	10.706
${Age}_{A}$	−0.267 ***	−5.341	−0.250 ***	−5.182	−0.315 ***	−6.345
${Age}_{B}$	−0.352 ***	−7.654	−0.338 ***	−7.610	−0.333 ***	−7.363
${\mathrm{MAP}}_{A}$	0.309 ***	2.032	0.221	1.512	0.291 *	1.921
${\mathrm{MAP}}_{B}$	0.275 **	1.993	0.221 *	1.657	0.236 *	1.709
${\mathrm{SAP}}_{A}$	−0.031	−0.237	−0.010	−0.078	−0.153	−1.170
${\mathrm{SAP}}_{B}$	−0.441 ***	−3.491	−0.422 ***	−3.342	−0.304 **	−2.504
${\mathrm{JMC}}_{A}$	−0.081	−0.713	−0.036	−0.325	0.072	0.647
${\mathrm{JMC}}_{B}$	0.278 ***	2.630	0.328 ***	3.208	0.184 *	1.797
*N*	759		759		759	
Log-likelihood	−4139		−4195.7		−4206.7	
McFadden R square	0.106		0.094		0.091	

Note: One, two, three and four stars (*) indicate respectively significance at the 10%, 5%, 1% and 0.5% level. Variable subscripts in ordered-RPL model denote choice mode: A = alternative A: relatively mild illness with low prevention price; B = alternative B: relatively sever illness with high prevention price.

**Table 5:** Heterogeneous effects of perceived health risk on choice behavior, by proximity to pollution source.

1	2	3	4	5	6	7
Variables	Lightly Polluted Areas	T-Value	Moderate Polluted Areas	T-Value	Severely Polluted Areas	T-Value
Acute upper respiratory tract infection * ln(duration + 1) (AI*LD)	0.091	-	0.175	-	0.087	-
Acute Bronchitis * ln(Duration + 1) (AB*LD)	−0.051	−1.4435	−0.069	−1.0497	−0.055	−0.8611
SD	0.000	0.0000	0.001	0.0002	0.000	0.0001
Acute Pneumonia * ln(Duration + 1) (AP*LD)	−0.040	−1.0864	−0.106	−1.5615	−0.032	−0.4920
S.D	0.478 **	10.7158	0.589 ***	6.6072	0.462 ***	5.9455
No Limitation (NL)	0.582	-	0.683	-	0.918	-
At home (AH)	−0.007	−0.0091	−0.087	−0.0639	−0.073	−0.0578
S.D	0.000	0.0001	0.003	0.0004	0.007	0.0009
In hospital (IH)	−0.575 ***	−4.3801	−0.596 ***	−2.7580	−0.845 ***	−3.0156
S.D	1.441 ***	6.1734	1.667 ***	4.2456	2.051 ***	5.2880
*Price*	0.033 ***	3.8242	0.046 ***	2.5976	0.034 **	2.1298
$Exposure_{A}$	0.338 ***	5.3933	0.472 ***	3.5962	−0.138	−0.9936
$Exposure_{B}$	0.188 ***	3.5165	0.492 ***	4.6626	−0.072	−0.5175
$Exposure_{A}^{2}$	−0.174 ***	−7.5286	−0.208 ***	−5.4870	−0.028	−0.3514
$Exposure_{B}^{2}$	−0.068 ***	−3.4167	−0.185 ***	−5.7982	0.046	0.5995
$Hazardousness_{A}$	−0.208 ***	−4.2627	−0.245 *	1.6711	0.150	1.2373
$Hazardousness_{B}$	−0.065	−1.5533	−0.332 **	−2.4187	0.108	0.9257
$Hazardousness_{A}^{2}$	−0.065 ***	−4.0336	−0.050	−0.6199	0.077	1.0756
$Hazardousness_{B}^{2}$	−0.013	−0.9055	−0.051	−0.6646	0.051	0.7804
$Exposure\times Hazardousness_{A}$	0.230 ***	6.1769	0.103	1.6321	−0.163	−1.5038
$Exposure\times Hazardousness_{B}$	0.138 ***	4.6727	−0.007	−0.1060	−0.136	−1.2492
${\mathrm{Family}\;\mathrm{health}}_{A}$	−0.079	−0.5857	−0.616 *	−1.7600	1.745 ***	5.0478
${\mathrm{Family}\;\mathrm{health}}_{B}$	0.268 **	2.1420	−0.275	−0.8729	2.626 ***	7.5509
${\mathrm{Family}\;\mathrm{size}}_{A}$	−0.312 ***	−3.8581	0.151	0.5283	−0.902 ***	−3.4883
${\mathrm{Family}\;\mathrm{size}}_{B}$	−0.242 ***	−3.2633	0.148	0.5275	−0.887 ***	−3.1262
${\mathrm{Income}}_{A}$	0.002	0.0319	−0.352 **	−2.1124	−0.314 **	−2.2428
${\mathrm{Income}}_{B}$	0.151 **	2.6563	−0.242 *	−1.6921	−0.153	−1.1751
${\mathrm{Education}}_{A}$	0.269 ***	6.0633	0.395 ***	3.5805	0.349 ***	3.4411
${\mathrm{Education}}_{B}$	0.392 ***	9.3348	0.422 ***	4.0159	0.836 ***	8.1224
$Age_{A}$	−0.264 ***	−4.4248	−0.396 ***	−2.6258	−0.083	−0.4215
${Age}_{B}$	−0.310 ***	−5.6574	−0.434 ***	−2.9395	−0.353 **	−2.0753
${\mathrm{JMC}}_{A}$	−0.20321	−1.3685	−1.357 ***	−3.4844	1.415 ***	5.0928
${\mathrm{JMC}}_{B}$	0.230096 *	1.6857	−1.083 ***	−2.9658	2.034 ***	7.1594
*N*	220		221		318	
Log-likelihood	−2488.4		−773.87		−743.78	
McFadden R square	0.110		0.140		0.184	

Note: One, two and three stars (*) indicate respectively significance at the 10%, 5%, and 1% level.

**Table 6:** Average WTP estimates by disease, activity restriction and duration (RMB per year).

1	2	3	4	5
Disease	Duration	Activity Restriction Level		
		No Limitation	At Home	In Hospital
Acute upper respiratory tract infection (AI)	5	0	0	-
	9	0	0	-
	15	0	0	-
Acute bronchitis (AB)	5	0	3.833	19.551
	9	0	4.619	20.337
	15	0	5.342	21.060
Acute pneumonia (AP)	5	0	3.468	19.186
	9	0	4.150	19.868
	15	0	4.778	20.495

## Appendix A. Unordered Choice Sets

**Table A1:** Choice set 1.

Alternative	A	B	C
Disease	Acute Pneumonia	Acute Pneumonia	I don’t want to purchase either
Duration	9-day episode	15-day episode	
Daily activity	No Limitation	No Limitation	
Price	300 RMB	500 RMB	
Which alternative do you prefer to purchase			

**Table A2:** Choice set 2.

Alternative	A	B	C
Disease	Acute Bronchitis	Acute Bronchitis	I don’t want to purchase either
Duration	5-day episode	5-day episode	
Daily activity	At home	In Hospital	
Price	300 RMB	500 RMB	
Which alternative do you prefer to purchase			

**Table A3:** Choice set 3.

Alternative	A	B	C
Disease	Acute upper respiratory tract infection	Acute Pneumonia	I don’t want to purchase either
Duration	9-day episode	5-day episode	
Daily activity	At home	No Limitation	
Price	300 RMB	100 RMB	
Which alternative do you prefer to purchase			

**Table A4:** Choice set 4.

Alternative	A	B	C
Disease	Acute upper respiratory tract infection	Acute Bronchitis	I don’t want to purchase either
Duration	5-day episode	15-day episode	
Daily activity	At home	In Hospital	
Price	300 RMB	500 RMB	
Which alternative do you prefer to purchase			

**Table A5:** Choice set 5.

Alternative	A	B	C
Disease	Acute Pneumonia	Acute Bronchitis	I don’t want to purchase either
Duration	9-day episode	15-day episode	
Daily activity	No Limitation	No Limitation	
Price	100 RMB	300 RMB	
Which alternative do you prefer to purchase			

**Table A6:** Choice set 6.

Alternative	A	B	C
Disease	Acute Bronchitis	Acute Pneumonia	I don’t want to purchase either
Duration	9-day episode	5-day episode	
Daily activity	At home	At home	
Price	500 RMB	300 RMB	
Which alternative do you prefer to purchase			

## Appendix B. Ordered Choice Sets

**Table A7:** Choice set 1.

Alternative	A	B	C
Disease	Acute Pneumonia	Acute Pneumonia	I don’t want to purchase either
Duration	9-day episode	15-day episode	
Daily activity	No Limitation	No Limitation	
Price	300 RMB	500 RMB	
Which alternative do you prefer to purchase			

**Table A8:** Choice set 2.

Alternative	A	B	C
Disease	Acute Bronchitis	Acute Bronchitis	I don’t want to purchase either
Duration	5-day episode	5-day episode	
Daily activity	At home	In Hospital	
Price	300 RMB	500 RMB	
Which alternative do you prefer to purchase			

**Table A9:** Choice set 3.

Alternative	A	B	C
Disease	Acute Pneumonia	Acute upper respiratory tract infection	I don’t want to purchase either
Duration	5-day episode	9-day episode	
Daily activity	No Limitation	At home	
Price	100 RMB	300 RMB	
Which alternative do you prefer to purchase			

**Table A10:** Choice set 4.

Alternative	A	B	C
Disease	Acute upper respiratory tract infection	Acute Bronchitis	I don’t want to purchase either
Duration	5-day episode	15-day episode	
Daily activity	At home	In Hospital	
Price	300 RMB	500 RMB	
Which alternative do you prefer to purchase			

**Table A11:** Choice set 5.

Alternative	A	B	C
Disease	Acute Pneumonia	Acute Bronchitis	I don’t want to purchase either
Duration	9-day episode	15-day episode	
Daily activity	No Limitation	No Limitation	
Price	100 RMB	300 RMB	
Which alternative do you prefer to purchase			

**Table A12:** Choice set 6.

Alternative	A	B	C
Disease	Acute Pneumonia	Acute Bronchitis	I don’t want to purchase either
Duration	5-day episode	9-day episode	
Daily activity	At home	At home	
Price	300 RMB	500 RMB	
Which alternative do you prefer to purchase			
